# Prison and opportunities for the management of COVID-19

**DOI:** 10.3399/bjgpopen20X101106

**Published:** 2020-06-10

**Authors:** Des Crowley, Walter Cullen, Patrick O'Donnell, Marie Claire Van Hout

**Affiliations:** 1 Assistant Director, Management of Addiction in Primary Care Programme, Irish College of General Practitioners, Dublin, Republic of Ireland; 2 Senior Lecturer, School of Medicine, University College Dublin, Dublin, Republic of Ireland; 3 Professor of Urban General Practice, Department of Health Sciences, University College Dublin College of Health Sciences, Dublin, Republic of Ireland; 4 General Practitioner and Clinical Fellow in Social Inclusion, UL Graduate Entry Medical School, University of Limerick, Limerick, Republic of Ireland; 5 Professor of Public Health Policy and Practice, Liverpool John Moores University, Liverpool, UK

**Keywords:** Prisoner, Prison, COVID-19, Coronavirus, General practice, Primary health care

COVID-19 is a new highly contagious viral infection that can be transmitted by asymptomatic patients. Without a vaccine or a specific cure for this disease, our current management strategy is to attempt to contain its spread. The many risks and challenges of managing COVID-19 in prisons have been reported and include: overcrowded prison environments, the poor general heath profile of prisoners, the quality of prison healthcare services, existing high levels of communicable diseases, and the inability to comply with most social distancing and hand hygiene rules.^1^ However, little attention has been focused on the opportunities that prison healthcare can provide in the management of COVID-19 and other public health concerns.

## Opportunities for improved prison health care

The majority of the prison population globally (estimated at 11 million people) are young adult males, a cohort not typically considered vulnerable to the serious effects of COVID-19.^2^ Prison life is based on structure and routine, factors which may contribute to effective management of infection outbreaks.

### Screening and prevention

Over 30 million people worldwide come into prisons’ health services annually.^2^ Prison committal is an opportunity to review prisoners’ health needs, and has been identified as an opportunity to screen for infections.^3^ A previous systematic review highlighted the success of opt-out committal HIV and hepatitis C screening in prison settings.^3^

Prison committal provides an opportunity to screen patients for COVID-19, and should be part of any containment strategy involving increased testing and contact tracing. People entering and exiting prison are some of the most vulnerable and underserved by community health services. They experience stigma and discrimination, creating barriers to accessing health care, and incarceration could be an opportunity to address unmet health needs.^4,5^ These may include screening and treatment for mental health problems, communicable diseases, and substance use disorders.^4^ Incarceration also provides a window for health promotion, self-care, improving health literacy, and disease prevention.^5^ Prisons are also locations where large numbers of people can be offered vaccinations, and this will hopefully form a critical part of the long-term strategy for the management of COVID-19.

### Delivery of prison health care

Many prisoners have complex medical and psychosocial needs that are rarely addressed fully while incarcerated.^4,5^ There have been many reports of new approaches and rapid adaptation of telemedicine to manage the risks of COVID-19 transmission in community settings.^6,7^ General practice has been at the forefront of these changes, and GPs are the main providers of routine health care in prisons globally.^5,7^ There have been no reports in the published literature of such innovation in the delivery of health care in prisons. While acknowledging the challenges of adopting telemedicine in prison settings, it would seem like an appropriate and efficient way to deliver health care to large groups of prisoners. It also could have the added benefit of giving prisoners access to the opinions of medical specialists in secondary care, while reducing the requirement to attend hospital services including accident and emergency departments. This would reduce cost, free up staff, and reduce the risks of spreading COVID- 19 both in prison and community settings.

### Drug use and policy

Early release of certain groups of prisoners to reduce prison populations worldwide has been recommended by the World Health Organization (WHO) and other international organisations in recent months.^1^ For years, public health specialists and experts on international drug policy have called for the implementation of less punitive and more human rights- and harm reduction-based drug policies to reduce the incarceration of drug users.^8^ The concentration of communicable infections in prisons is mainly due the incarceration of people who inject drugs (PWID).^4^ PWID acquire HIV and hepatitis C virus in the community and in prison settings, and lack of access to evidence-based harm reduction measures — such as opioid agonist treatment (OAT), and needle and syringe programmes — is a major concern, particularly in prison settings.^9,10^ There is now an opportunity to revisit this deficit and use the present focus on COVID-19 management to advocate for increased harm reduction services and the decriminalisation of drug users as measures to reduce present and future communicable infections.

## Challenges to improved prison health care

There is great variation in prison healthcare standards globally.^4^ Systematic neglect and underfunding of these services in many countries has resulted in prison health services that lack ambition, oversight, and links to public health.^4,5^

### Transitioning back to the community

Reducing prison populations is a critical aspect of the management of COVID-19 in prisons. However, it must be recognised that the period immediate after release is considered high risk and is associated with high levels of deaths from overdose.^10,11^ Maintaining prisoners on OAT as they transition back to the community is protective, but it needs planning and organisation. Many prisoners experience homelessness, and releasing them back into the community will increase their risk of acquiring COVID-19. Community drug users will experience the same risks as the general population, and will have enhanced risks due to their own vulnerability and through drug use itself.^12^ Older drug users and those with underlying health conditions have even more complex vulnerabilities.^12^ It is important that any decisions made to release prisoners involves a risk assessment, and release is planned and supported to ensure that it does not increase their risk of contracting COVID-19.^12^

### Public health and human rights

There have been numerous calls for increased involvement of public health specialists in prison health care, and it is recognised that improving the health of prisons will ultimately improve the health of the wider community.^4,5^ Prisons are dynamic institutions with the daily movement of prisoners, staff, and visitors between prison and the community. They are a place of work for many, including healthcare providers. Most prisoners return to their communities.

Prisoners should enjoy the same standards of health care that are available in the community, and their rights should be safeguarded according to international treaties.^8^ Prison and state authorities should not use the management of COVID-19 and potential restrictions to undermine the rights of prisoners, and any COVID-19 related initiatives have to be underpinned by these accepted principles. The WHO are leading the global public health response to COVID-19. Their recommendations are being implemented in large numbers of countries worldwide. A unified and structured public health approach is crucial to achieving influence in advising on national health policy. This is a once in a generation opportunity to embed prison health into public health, and it should not be missed

## Conclusion

The current pandemic provides an unprecedented opportunity to review the purpose of incarceration, historically seen as punishment and rehabilitation. Our treatment of people while in custody should be safe, secure, and humane. Social inequalities are a major driver of prison populations. Improving social and health inequalities could improve the lives of so many into the future. As we adjust to and manage the economic impact of COVID-19, we must strive for a safer and humane custody, underpinned by a human rights agenda and a realisation of the duty of care that society has to those deprived of their liberty.

## References

[1] United Nations Office on Drugs and Crime (UNODC) (2020). COVID-19 preparedness and responses in prisons. https://www.unodc.org/documents/justice-and-prison-reform/UNODC_Position_paper_COVID-19_in_prisons.pdf.

[2] World Prison Brief (2020). An online database comprising information on prisons and the use of imprisonment around the world. http://prisonstudies.org/.

[3] European Centre for Disease Prevention and Control, European Monitoring Centre for Drugs and Drug Addiction (2017). Systematic review on active case finding of communicable diseases in prison settings.

[4] Fazel S, Baillargeon J (2011). The health of prisoners. The Lancet.

[5] Enggist S, Møller L, Galea G, Udesen C, World Health Organization Regional Office for Europe (2014). Prisons and health.

[6] Zhou X, Snoswell CL, Harding LE (2020). The role of telehealth in reducing the mental health burden from COVID-19. Telemed J E Health.

[7] Greenhalgh T, Koh GCH, Car J (2020). Covid-19: a remote assessment in primary care. BMJ.

[8] United Nations (1990). Basic Principles for the Treatment of Prisoners. https://www.un.org/ruleoflaw/blog/document/basic-principles-for-the-treatment-of-prisoners/.

[9] Larney S, Peacock A, Leung J (2017). Global, regional, and country-level coverage of interventions to prevent and manage HIV and hepatitis C among people who inject drugs: a systematic review. Lancet Glob Health.

[10] Degenhardt L, Grebely J, Stone J (2019). Global patterns of opioid use and dependence: harms to populations, interventions, and future action. The Lancet.

[11] Merrall ELC, Kariminia A, Binswanger IA (2010). Meta-analysis of drug-related deaths soon after release from prison. Addiction.

[12] European Monitoring Centre for Drugs and Addiction (2020). EMCDDA publishes update on the implications of COVID-19 for people who use drugs and for drug service providers. https://www.emcdda.europa.eu/news/2020/emcdda-publishes-update-on-the-implications-of-COVID-19-for-people-who-use-drugs-and-for-drug-service-providers_en.

